# Effects of dietary canthaxanthin on egg production, serum parameters, and intestinal health in indigenous chickens under heat stress

**DOI:** 10.3389/fvets.2025.1672896

**Published:** 2025-10-03

**Authors:** Xiaoyun Zhou, Yiping Song, Jie Chen, Xi Chen, Lanxin Guan, Yaxuan Wang, Mei Xiao, Wenchao Liu, Lilong An

**Affiliations:** Department of Animal Science, College of Coastal Agricultural Sciences, Guangdong Ocean University, Zhanjiang, China

**Keywords:** canthaxanthin, high temperature, laying hens, small intestinal mucosa, nutrient absorption, egg production rate

## Abstract

High-temperature environments significantly compromise the productivity of laying hens by damaging intestinal mucosal structure and impairing nutrient absorption. The effects of dietary canthaxanthin (CX) supplementation on egg production rate and intestinal health in Huaixiang chickens raised at high temperatures were assessed in this study. Six groups were randomly selected from among 216 hens: NC (basal diet, 25 ± 1°C), HC (basal diet, 32 ± 1°C for 8 h/day), and four HCX groups (basal diet supplemented with 4, 6, 8, or 10 mg/kg CX, 32 ± 1°C for 8 h/day), with six replicates of six birds each over 28 days. High temperature significantly decreased feed intake, egg production rate, and feed conversion ratio (FCR; *p* < 0.05), reduced serum total antioxidant capacity (T-AOC), superoxide dismutase (SOD) and glutathione peroxidase (GSH-Px) activities (*p* < 0.05), while increasing malondialdehyde (MDA) and reactive oxygen species (ROS; *p* < 0.05). High temperature also decreased T-AOC activity in the duodenum, jejunum and ileum (*p* < 0.05), and increased MDA and ROS levels in these intestinal segments (*p* < 0.05). Relative to the HC group, dietary CX increased egg production rate and FCR, enhanced serum T-AOC, SOD and GSH-Px activities, while reducing MDA and ROS levels (*p* < 0.05). CX increased T-AOC activity in the small intestine and decreased MDA and ROS levels (*p* < 0.05). In addition, heat stress impaired intestinal morphology, lowering villus height (VH), villus surface area (VSA), and villus height to crypt depth ratio (V/C; *p* < 0.05) while increasing apoptosis rate (*p* < 0.05). This was accompanied by decreased jejunal fatty acid binding protein 1 (*FABP1*) expression and lowered serum concentrations of total protein (TP), total cholesterol (TC), triglycerides (TG), low-density lipoprotein cholesterol (LDL-C), and high-density lipoprotein cholesterol (HDL-C; *p* < 0.05). Relative to the HC group, dietary CX alleviated intestinal villus atrophy and rupture, effectively maintained normal small intestinal VH, VSA, and V/C ratios, and significantly reduced intestinal epithelial cell apoptosis rate. CX significantly increased serum TP, TG, TC, LDL-C, and HDL-C while maintaining normal expression levels of *FABP1* mRNA in the jejunum. These results demonstrate that dietary supplementation with 8 mg/kg CX effectively mitigates high temperature-induced declines in egg production by improving intestinal nutrient absorption.

## 1 Introduction

With the intensification of global warming, the impact of high-temperature environments on poultry production has become increasingly prominent, especially in tropical and subtropical areas. Heat stress is now a major factor limiting the poultry industry's development. High temperatures not only reduce poultry feed intake but also damage intestinal structure and function, resulting in decreased nutrient digestion and absorption efficiency, ultimately affecting laying performance (1). Studies have shown that heat stress can lead to an excessive accumulation of reactive oxygen species (ROS) in the intestine, attacking membrane phospholipids, mitochondria, and DNA structures of intestinal epithelial cells, causing oxidative damage (2, 3). This is often manifested as increased crypt depth, decreased villus height, and reduced villus height/crypt depth ratio (V/C), subsequently affecting nutrient absorption function (4). Meanwhile, heat stress further inhibits the absorption of small peptides and lipids by downregulating the mRNA expression of small peptide transporter (*PepT1*) and fatty acid binding protein (*FABP1*) (5). These pathological changes directly cause a significant decrease in the egg production rate of laying hens. Therefore, developing efficient antioxidants to alleviate heat stress-induced intestinal injury and improve nutrient absorption rates has become the focus of current poultry nutrition research.

Canthaxanthin (CX), a red-orange ketone carotenoid chemically named β, β-carotene-44′-dione (C40H5_2_O_2_), is widely used in feed, medicine, and food industries, primarily as a colorant (6). However, beyond its coloring property, its potent antioxidant activity has garnered increasing interest in recent years. Distinct from many common carotenoids, CX possesses two ketone groups on its terminal rings. This unique structure enhances its electron-accepting capacity, thereby conferring superior free radical scavenging and antioxidant activities compared to its counterparts (7). Although the antioxidant properties of carotenoids like lycopene and astaxanthin have been relatively well-documented in heat-stressed poultry (8, 9), the potential of CX, especially in laying hens, remains underexplored. A previous study demonstrated that dietary CX supplementation at 6 mg/kg significantly enhanced serum total antioxidant capacity (T-AOC) in broiler breeders under thermoneutral conditions (10). However, its efficacy in alleviating heat stress-induced intestinaloxidative damage and thereby improving production performance in heat-susceptible laying hens remains unclear. The Huaixiang chicken, an indigenous breed prevalent in southern China's tropical and subtropical regions, serves as an ideal model for such investigation. This breed is characterized by low heat tolerance and a narrow optimal temperature range of 18–24°C. Exposure to ambient temperatures exceeding 28°C consistently triggers significant declines in egg production rate in this breed, mirroring the severe challenges faced by the poultry industry in hot climates.

This study aims to explore the effects of CX on intestinal morphology, antioxidant function, and nutrient absorption of Huaixiang chickens under high temperature environment. By examining morphological alterations in the small intestinal mucosa, apoptosis levels, antioxidant enzyme activities, and absorptive cell structures, along with analyzing mRNA expression of nutrient transporters (*PepT1* and *FABP1*) and the levels of serum total protein and lipids, the molecular mechanism by which CX alleviates intestinal injury caused by heat stress was systematically revealed. The results of this experiment will provide a theoretical basis for the application of CX as a functional feed additive in high-temperature poultry production while providing new Insights for developing nutritional strategies to enhance heat stress resistance.

## 2 Materials and methods

### 2.1 Experimental animals and drugs

Three hundred 26-week-old laying hens were acquired from Guangzhou Xiying Rare Poultry Breeding Co., Ltd. (Foshan, China). Canthaxanthin (CX, product code UE01611012, 10% purity) was supplied by DSM Vitamins Trading Co., Ltd. (Shanghai, China).

### 2.2 Experimental design, grouping and diet

This research was carried out in the poultry housing facility of Guangdong Ocean University's Animal Hospital (located in Zhanjiang, China). All protocols involving animals were reviewed and approved by the Institutional Animal Ethics Committee of the same institution. Environmental conditions, including temperature and humidity levels, were maintained using heating lamps and dehumidifiers. The normal temperature group (25 ± 1°C) and the high temperature group (cyclic high temperature of 32 ± 1°C for 8 h daily from 9:00 to 17:00) were established, with humidity maintained at 55%−70% in each group. The trial consisted of a 14-day pre-trial period (weeks 26–27) and a 28-day formal trial period (weeks 28–31). During the pre-trial period, all chickens were fed the basal diet under uniform conditions without selection. Six groups were randomly assigned to 216 healthy 26-week-old Huaixiang hens: the NC group (25 ± 1°C, basal diet), the HC group (32 ± 1°C for 8 h/day, basal diet), and four HCX groups (32 ± 1°C for 8 h/day, basal diet supplemented with 4, 6, 8, or 10 mg/kg CX). The specific grouping is shown in Table 1. The four supplementation levels of canthaxanthin (4, 6, 8, and 10 mg/kg) were selected to establish a comprehensive dose-response relationship. The central dose of 6 mg/kg was chosen based on its proven efficacy in enhancing the antioxidant status of poultry as reported by Zhang et al. (10). The lower (4 mg/kg) and higher (8, 10 mg/kg) doses were included to identify potential sub-optimal, optimal, and plateau effects. All experimental doses were well below the maximum authorized level of 30 mg/kg for laying hens stipulated in Commission Regulation (EU) 2015/1486 (11), ensuring both safety and regulatory compliance. All animal feeds were formulated according to the Chinese agricultural industry standard NY/T 33-2004 for poultry nutrition requirements, with the primary ingredients being corn and soybean-based protein sources. The detailed feed composition is provided in Table 2.

**Table 1:** Arrangement of the treatments.

**Group**	**Abbreviation**	**Diet**
Normal temperature control group	NC	Basic diet
High temperature control group	HC	Basic diet
High-temperature canthaxanthin 4 mg/kg group	HCX4	Basic diet +4 mg/kg CX
High-temperature canthaxanthin 6 mg/kg group	HCX6	Basic diet +6 mg/kg CX
High-temperature canthaxanthin 8 mg/kg group	HCX8	Basic diet +8 mg/kg CX
High-temperature canthaxanthin 10 mg/kg group	HCX10	Basic diet +10 mg/kg CX

**Table 2:** Basal diet composition and nutrient level (air-dried basis).

**Items**	**Content**
Corn	55.00
Soybean meal	20.00
Wheat bran	9.50
Fish meal	5.00
Limestone	7.50
CaHPO4	2.50
NaCl	0.10
Premix^1^	0.40
Total	100.00
ME/(MJ/kg)^2^	11.60
CP	15.50
Ca	2.0
TP	0.63
Met	0.40
Cys	0.30
Lys	0.80

The nutrient levels are calculated values. ^1^The premix provided the following per kilogram of diet: vitamin A 9000 IU, vitamin D 2500 IU, vitamin E 20 IU, vitamin B12 12 μg, vitamin K 2.4 mg; Microelements: Mn 100 mg, Zn 60 mg, Fe 25 mg, Cu 5 mg, Co 0.1 mg (sulfates form); Se(Na_2_SeO_3_) 0.2 mg, I (KI) 0.5 mg. ^2^Except for metabolic energy (ME), others are measured values.

### 2.3 Acquisition of samples

At the end of the 28-day experiment, three hens per replicate, amounting to 18 birds per group and 108 birds in total, were randomly selected after a fasting period. From each bird, ~5 ml of blood was collected using serum separation gel tubes. The samples were centrifuged, and the resulting serum was aliquoted and stored at −20°C pending analysis. Following blood collection, chickens were euthanized by cervical dislocation performed by trained personnel (12, 13). Death was confirmed by absence of corneal reflex and cessation of breathing for >3 min. Approximately 1 cm tissue sections from the duodenal, jejunal, and ileal regions were then excised for analysis. These segments were washed with ice-cold phosphate-buffered saline (1 × PBS) and immersion-fixed in 4% paraformaldehyde after luminal clearance.

### 2.4 Egg production rate, average daily feed intake, and feed conversion ratio (FCR)

Throughout the trial period, the egg production rate, average daily feed intake (ADFI), and feed conversion ratio (FCR) of each group were recorded.

Egg production rate (%) = egg production per replicate per day/number of chickens in the replicate.

ADFI (g/bird) = feed intake per replicate per day/number of chickens in the replicate.

Total feed consumption per replicate was recorded over the entire experiment. Total egg mass was the sum of the weight of all eggs collected per replicate throughout the period. The FCR was calculated using the following formula: the FCR (kg feed/kg egg) = total feed consumption (kg)/total egg mass (kg).

### 2.5 Detection of serum biochemical parameters

Serum concentrations of total protein (TP), triglycerides (TG), total cholesterol (TC), high-density lipoprotein cholesterol (HDL-C), and low-density lipoprotein cholesterol (LDL-C) were measured using commercial ELISA kits (Jiangsu Meimian Industrial Co., Ltd., Jiangsu, China) (14).

### 2.6 Observation of intestinal histological morphology

The fixed small intestinal tissues were made into paraffin sections and stained with HE by Wuhan Saville Biotechnology Co., Ltd., and the results were observed and preserved. After taking photos, the villus height (VH), villus width (VW), and crypt depth (CD) were quantified using ImageJ software (v1.44). There were 6 replicates in each group. Three discontinuous slices were randomly selected for each replicate, with 10 intact villi per slice selected to measure VH, VW, and CD values. The villus height-to-crypt depth ratio (V/C) was then computed (15). The villus surface area was estimated using the formula: VSA = 2π × (VW/2) × VH, where VW represents villus width measured at the midpoint of the villus (16).

### 2.7 Observation on the microstructure of intestinal mucosal epithelial cells

Approximately 2 mm segments of duodenum, jejunum, and ileum were collected, and the extraneous fat was quickly removed before fixation in electron microscopy fixative at room temperature for 2 h, followed by storage at 4°C. The samples were transported to Wuhan Borf Biotechnology Co., Ltd. for preparation of ultrathin sections and subsequent observation of small intestinal mucosal epithelial cell microstructure by transmission electron microscopy.

### 2.8 Observation of fluorescence apoptosis in small intestinal tissue

To determine the percentage of apoptotic cells in the small intestine, three replicates were selected in each group. Three sections from each replicate were subjected to TUNEL assay using the Fluorescein *in Situ* Apoptosis Detection Kit (Beyotime, Shanghai, China). For 1 h at 37°C, the intestinal sections were incubated in acetate buffer containing precursors labeled with fluorescein (FITC) and terminal deoxynucleotidyl transferase (TdT). Following incubation, tissue sections underwent three washes with PBS supplemented with 0.05% Tween-20. Nuclei were stained using 4′,6-diamidino-2-phenylindole (DAPI; Invitrogen, Carlsbad, CA) as a counterstain, after which samples were coverslipped using ProLong Gold Antifade Mountant (Invitrogen). To validate the TUNEL assay's specificity, the TdT enzyme and probe combination was substituted with acetate buffer. Cellular nuclei and TUNEL-positive signals were visualized through blue (DAPI) and green (FITC) fluorescence channels, respectively. Fluorescence microscopy was employed for observation and image acquisition, with TUNEL-positive cell percentages in intestinal tissue being calculated using ImageJ software (v1.44) (17).

### 2.9 Detection of antioxidant indicators in the small intestine

Approximately 1 g of small intestinal tissue was weighed and homogenized with 10% PBS solution (1:9, wt/vol), and then centrifuged at 2,000–3,000 rpm for 20 min to collect the supernatant. Total antioxidant capacity (T-AOC), malondialdehyde (MDA), and reactive oxygen species (ROS) in duodenal, jejunal, and ileal tissues were analyzed using commercial ELISA kits (Meimian Industrial, China) (18). The absorbance for all these intestinal antioxidant parameters was measured at a wavelength of 450 nm using a Synergy H1 multifunctional microplate reader (BioTek Instruments, USA).

### 2.10 Detection of serum antioxidant indicators

Commercial assay kits provided by the manufacturer (Meimian Industrial, China) were used to quantify the following serum oxidative stress parameters: reactive oxygen species (ROS) levels, glutathione peroxidase (GSH-PX) activity, superoxide dismutase (SOD) activity, malondialdehyde (MDA) content, and total antioxidant capacity (T-AOC). The absorbance was measured at 450 nm wavelength (which was used for all the mentioned serum antioxidant parameters according to the kit instructions) using a Synergy H1 multifunctional microplate reader (BioTek Instruments, USA) (19, 20). The experiment strictly followed the kit operation specification, and all data were verified by three independent repeated experiments.

### 2.11 Relative mRNA expression analysis of nutrient transporters in jejunum

Jejunum samples weighing 50 mg were transferred into nuclease-free homogenization tubes (2.5 ml capacity), followed by the addition of 500 μl cell lysis solution (EZB-RN001, EZBioscience, USA). After complete lysis, RNA was purified through chromatography columns, washed, eluted, and extracted (Vazyme Biotech Co., Ltd., Jiangsu, China). RNA purity was assessed by measuring the absorbance ratio (A260/A280) using a spectrophotometer. Reverse transcription was performed with a commercial kit (Vazyme Biotech Co., Ltd.) to synthesize cDNA (21). Gene-specific primers (sequences shown in Table 3) were synthesized by Guangzhou Ruijie Biological Co., Ltd. Gene expression levels were quantified through quantitative real-time PCR (qPCR) analysis in 20 μl reactions containing synthesized cDNA templates. The thermal cycling protocol included an initial 2-min denaturation at 95°C, followed by 15-s denaturation at 95°C and 30-s annealing at 60°C, for 40 cycles (Bio-Rad, Hercules, CA, USA). Three biological replicates were analyzed for each sample, with β-actin serving as the reference gene for normalization. Relative gene expression was determined by applying the comparative CT (2^−ΔΔCT^) calculation method.

**Table 3:** Primer sequences.

**Genes**	**Primer sequence**	**Product size (bp)**
*PepT1*	F: TGGGGACTTTGGAGCTATGC	171
	R: CCAGCAAGGAACATGCCAAC	
*FABP1*	F: GTGTGAGATGGAGCTGCTGA	127
	R: GGTGATGGTGTCTCCGTTGA	
β-actin	F: TGAACCCCAAAGCCAACAGA	108
	R: CAGAGGCATACAGGGACAGC	

PepT1, peptide transporter 1; FABP1, fatty acid-binding protein 1.

### 2.12 Data analysis

Data were checked for normality using the Shapiro-Wilk test and for homogeneity of variances using Levene's test. All parameters were then subjected to one-way analysis of variance (ANOVA). Where significant differences were found, multiple comparisons among groups were performed using Tukey's honestly significant difference (HSD) *post hoc* test to control the Type I error rate. All statistical analyses were performed using SPSS 26.0. Data are presented as the mean ± standard deviation, and a probability value of *p* < 0.05 was considered statistically significant. Draw the bar chart using GraphPad Prism 10.

## 3 Results

### 3.1 Production performance

As shown in Table 4, high temperature significantly compromised the production performance of laying hens. Relative to the NC group, the HC group showed a 15.6% reduction in average daily feed intake and a 23.8% decrease in egg production rate (*p* < 0.05), while the feed conversion ratio was increased by 27.3% (*p* < 0.05). Dietary supplementation with CX effectively ameliorated these adverse effects under heat stress. Compared to the HC group, CX supplementation significantly improved egg production rate and reduced FCR (*p* < 0.05). The 8 mg/kg CX dose demonstrated the best efficacy in the production performance, significantly increasing egg production by 23.8% and decreasing FCR by 15.3% compared to the HC group (*p* < 0.05). However, CX supplementation did not significantly ameliorate the heat-induced reduction in ADFI, as no differences were observed between the CX-supplemented groups and the HC group (*p* > 0.05).

**Table 4:** The effects of CX-supplemented diets on average daily feed intake, egg production rate, and feed conversion ratio under heat stress (*n* = 6).

**Item**	**NC**	**HC**	**HCX4**	**HCX6**	**HCX8**	**HCX10**	***p*-Value**
ADFI (g/bird)	109 ± 0.59^a^	92.1 ± 2.96^b^	90.0 ± 2.96^b^	94.3 ± 1.76^b^	95.3 ± 1.01^b^	92.1 ± 0.69^b^	< 0.001
Egg production rate (%)	65.0 ± 1.55^a^	49.5 ± 2.26^d^	57.0 ± 1.10^c^	59.8 ± 3.19^bc^	61.3 ± 1.37^ab^	58.8 ± 3.13^bc^	< 0.001
FCR (g feed/g egg)	2.97 ± 0.07^c^	3.78 ± 0.23^a^	3.34 ± 0.15^b^	3.28 ± 0.18^b^	3.2 ± 0.07^b^	3.31 ± 0.17^b^	< 0.001

ADFI, average daily feed intake; FCR, feed conversion ratio. Data are presented as “mean ± SD.” In the same row, values with no letter or the same letter superscripts mean no significant difference (p > 0.05), while values with different letter superscripts mean significant difference (p < 0.05).

### 3.2 Serum biochemical constituents

As indicated in Table 5, high temperature significantly compromised serum biochemical constituents. Relative to the NC group, the HC group showed 41.9, 23.4, 20.1, 24.0, and 27.4% reductions in serum TP, TG, TC, HDL-C, and LDL-C levels, respectively (*p* < 0.05). Dietary supplementation with CX effectively ameliorated these alterations. The 6 mg/kg CX dose showed the most pronounced effect on serum TP, increasing its level by 33.6% compared to the HC group (*p* < 0.05). Meanwhile, the 8 mg/kg CX dose demonstrated the best efficacy on serum lipids, significantly elevating TG by 35.6%, TC by 15.0%, HDL-C by 24.8%, and LDL-C by 29.1% relative to the HC group (*p* < 0.05). Notably, serum TG, HDL-C, and LDL-C levels in the HCX8 group were restored to levels showing no significant difference from the NC group (*p* > 0.05). However, serum TP levels in the HCX6 group remained significantly lower than those in the NC group (*p* < 0.05). These results demonstrate a partial restorative effect of CX on serum constituents under heat stress.

**Table 5:** The effects of CX-supplemented diets on the content of serum total protein and the concentrations of serum lipids under heat stress (*n* = 6).

**Item**	**NC**	**HC**	**HCX4**	**HCX6**	**HCX8**	**HCX10**	***p*-Value**
Serum TP (mg/ml)	24.1 ± 2.03^a^	14.0 ± 0.56^c^	13.1 ± 1.03^c^	18.7 ± 2.36^b^	17.6 ± 0.55^b^	14.7 ± 0.64^c^	< 0.001
Serum TG (μmol/ml)	2.27 ± 0.08^a^	1.74 ± 0.05^c^	1.82 ± 0.09^c^	2.02 ± 0.09^b^	2.36 ± 0.07^a^	1.82 ± 0.06^c^	< 0.001
Serum TC (μmol/ml)	5.43 ± 0.20^a^	4.34 ± 0.18^c^	4.48 ± 0.14^c^	4.86 ± 0.09^b^	4.99 ± 0.13^b^	4.56 ± 0.17^c^	< 0.001
Serum HDL-C (μmol/ml)	1.75 ± 0.04^a^	1.33 ± 0.04^e^	1.40 ± 0.05^de^	1.49 ± 0.06^c^	1.66 ± 0.05^b^	1.42 ± 0.03^cd^	< 0.001
Serum LDL-C (μmol/ml)	3.32 ± 0.08^a^	2.41 ± 0.33^d^	2.80 ± 0.08^c^	3.01 ± 0.06^bc^	3.11 ± 0.08^ab^	2.85 ± 0.05^bc^	< 0.001

TP, total protein; TG, triglycerides; TC, total cholesterol; HDL-C, high-density lipoprotein cholesterol; LDL-C, low-density lipoprotein cholesterol. Data are presented as “mean ± SD.” In the same row, values with no letter or the same letter superscripts mean no significant difference (p > 0.05), while values with different letter superscripts mean significant difference (p < 0.05).

### 3.3 Intestinal structure

**Figure 1:**
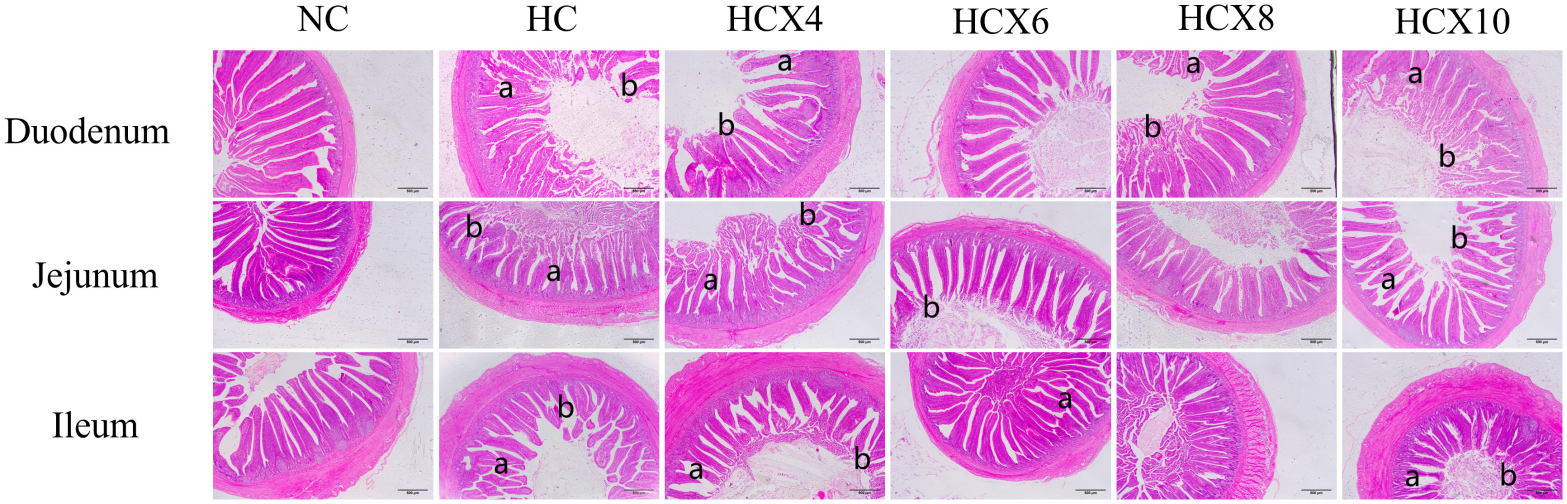
Effects of CX supplementation at varying concentrations on intestinal structure under heat stress (HE, 40×). Pathological features: **(a)** intestinal villus rupture; **(b)** intestinal villus atrophy.

As shown in Figure 1, high temperature caused significant damage to the mucosal structure of the duodenum, jejunum, and ileum, manifested through disordered villus arrangement, morphological deformation, and partial villus atrophy or rupture. The ileum showed the most pronounced villus rupture, followed by the jejunum. CX-supplemented diets under heat stress effectively alleviated mucosal structural damage in all three intestinal segments. Among them, the supplementation with CX at doses of 6 and 8 mg/kg had the best effect, and the villi were completer and more arranged neatly.

### 3.4 Intestinal morphology

**Figure 2:**
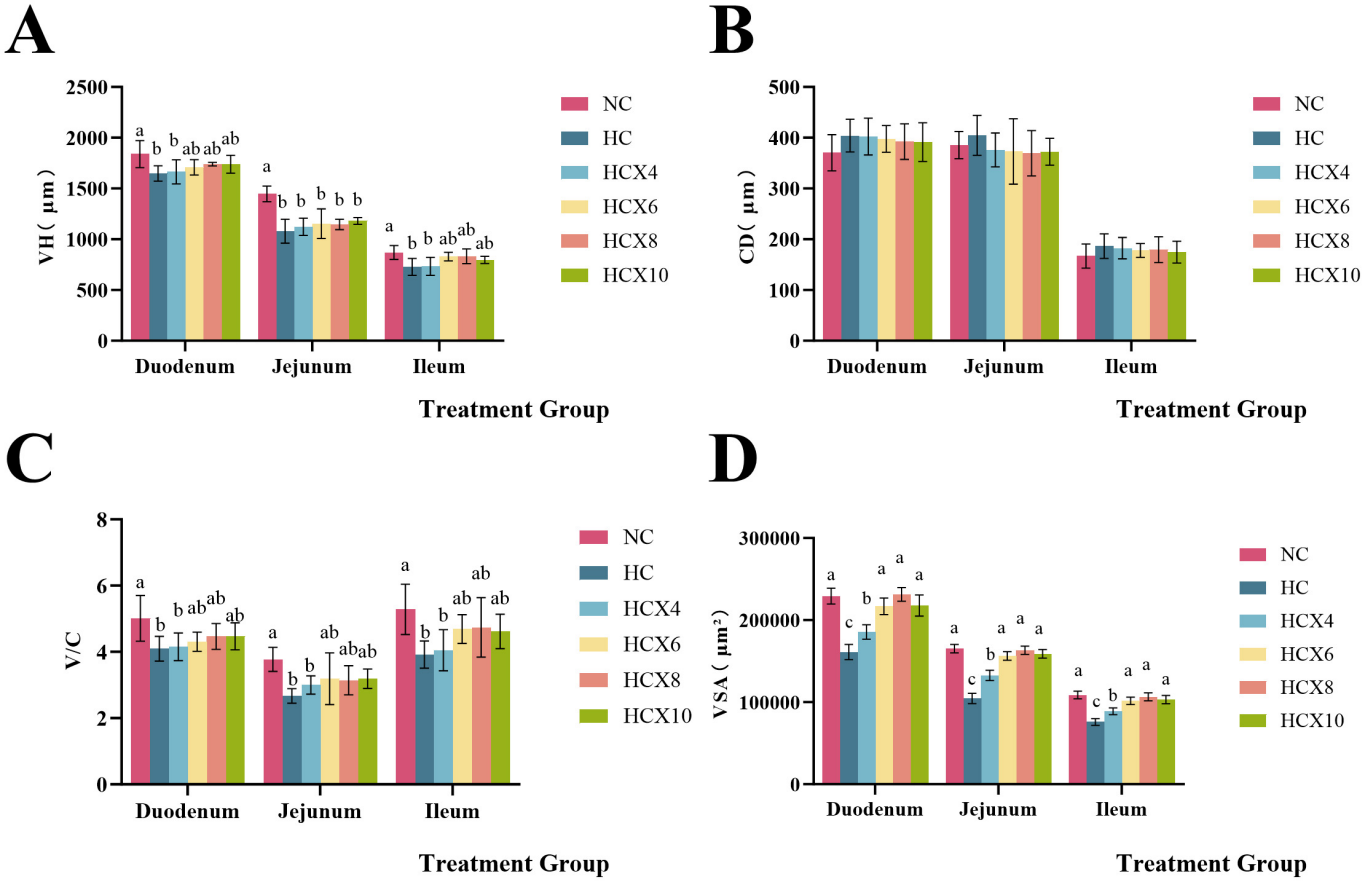
Effects of CX supplementation at varying concentrations on small intestine morphology under heat stress. **(A)** villus height (VH); **(B)** crypt depth (CD); **(C)** the ratio of villus height to crypt depth (V/C); **(D)** villus surface area (VSA). Values with different letters indicate significant differences (*p* < 0.05), while those with the same letters or no letters indicate no significant differences (*p* > 0.05).

As presented in Figure 2, high temperature significantly reduced both VH and V/C in the duodenum (*p* < 0.05), while CD remained unchanged (*p* > 0.05). The addition of 6, 8, and 10 mg/kg CX to the diet restored duodenal VH and V/C to normal temperature levels. In the jejunum, high temperatures significantly decreased VH and V/C (*p* < 0.05) without altering CD (*p* > 0.05), and dietary supplementation with 6, 8, and 10 mg/kg CX restored jejunal V/C to the normal temperature level. High temperature significantly reduced both VH and V/C in the ileum (*p* < 0.05), while CD remained unchanged (*p* > 0.05). The addition of 6, 8, and 10 mg/kg CX to the diet restored ileal VH and V/C to normal temperature levels. Furthermore, heat stress also significantly reduced the villus surface area (VSA) across all intestinal segments (*p* < 0.05). The addition of 6, 8, and 10 mg/kg CX to the diet effectively restored VSA in all three intestinal segments to normal temperature levels (*p* > 0.05).

### 3.5 Intestinal epithelial microstructure

**Figure 3:**
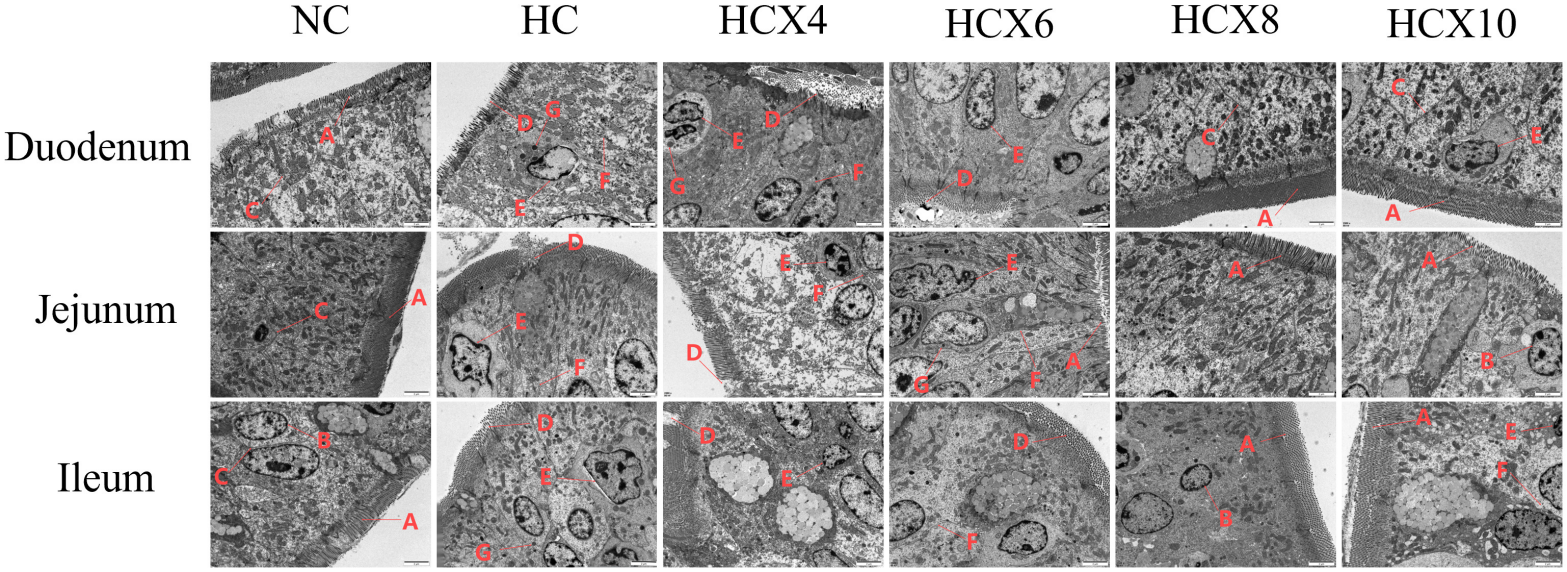
Effects of CX supplementation at varying concentrations on the microstructure of intestinal mucosal absorptive cells under heat stress (TEM, × 2,500). Pathological features: **(A)** microvilli arranged neatly, uniform length; **(B)** nuclear membrane without shrinkage; **(C)** clear cell membrane boundaries; **(D)** microvilli arranged irregularly, missing;**(E)** nuclear membrane deformation; **(F)** indistinct cell membrane boundaries; **(G)** apoptotic body.

As shown in Figure 3, high temperature induced the appearance of apoptotic bodies, nuclear membrane deformation, blurred cell membrane boundaries, and disorganized or even lost microvilli in small intestinal mucosal epithelial cells. In contrast, the CX group maintained relatively intact intestinal villous structures and mucosal integrity, with microvilli displaying regular arrangement and uniform length. The cell membrane boundaries were clearly defined, and nuclear membrane shrinkage and apoptotic body formation were significantly reduced.

### 3.6 Intestinal cell apoptosis

**Figure 4:**
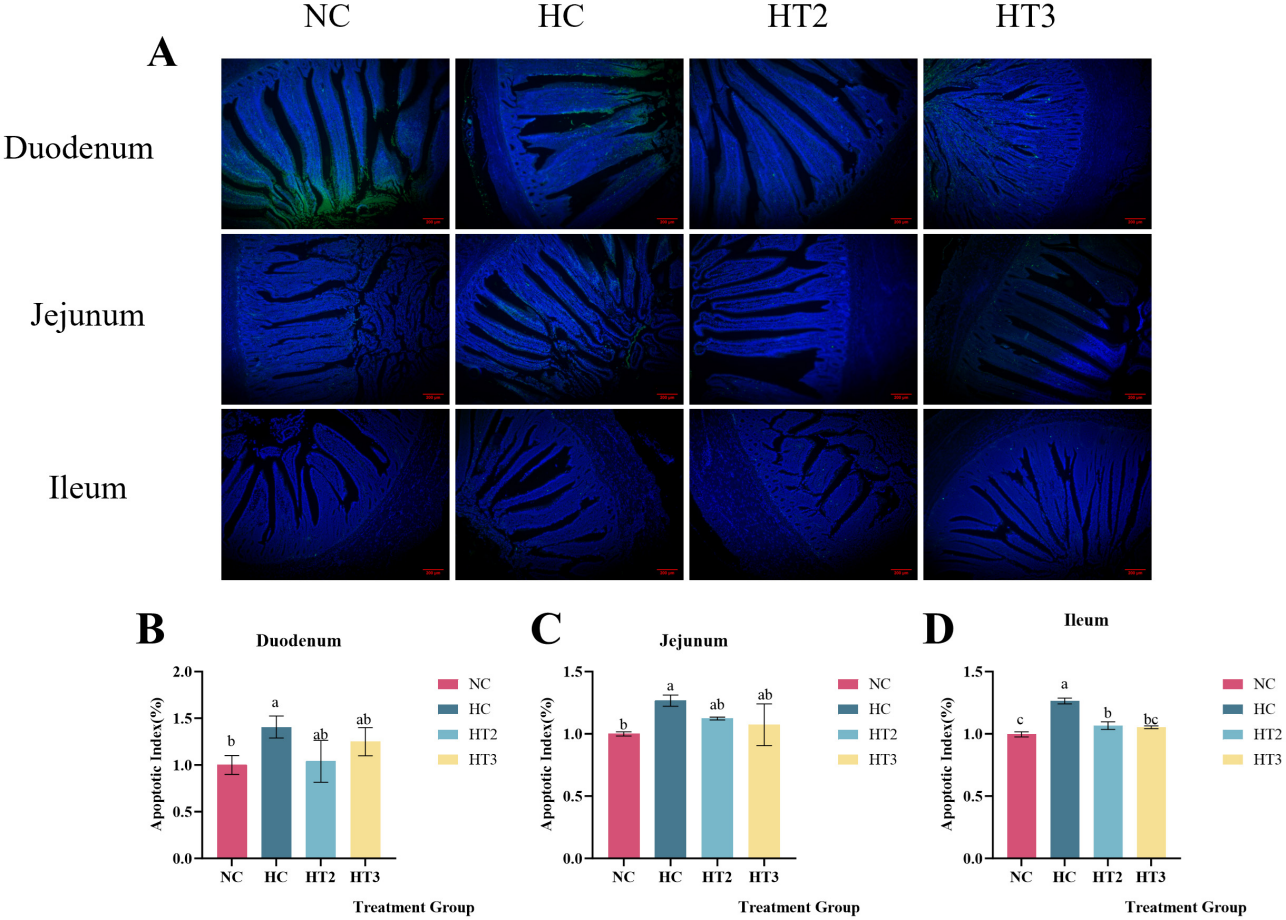
Effects of CX supplementation at varying concentrations on intestinal epithelial apoptosis: Duodenum **(A, B)**, Jejunum **(A, C)**, and Ileum **(A, D)** under heat stress. **(A)** Merged images of TUNEL (green) and DAPI (blue) staining in duodenal, jejunal, and ileal epithelial cells; **(B)** Quantitative analysis of the apoptotic cell ratio in the duodenum; **(C)** Quantitative analysis of the apoptotic cell ratio in the jejunum; **(D)** Quantitative analysis of the apoptotic cell ratio in the ileum. red scale bar = 200 μm. Values with different letters indicate significant differences (*p* < 0.05), while those with the same letters or no letters indicate no significant differences (*p* > 0.05).

The TUNEL method was used to detect the apoptosis rate of small intestinal cells in the NC, HC, HCX6, and HCX8 groups. As presented in Figure 4, relative to the NC group, high temperature significantly increased the apoptosis rate of epithelial cells in the duodenum, jejunum, and ileum (*p* < 0.05). Adding 6 and 8 mg/kg CX to the diet can restore the apoptosis rate of duodenal and jejunal cells to the level of the control group at normal temperature (*p* > 0.05), while 8 mg/kg CX can restore the apoptosis rate of ileal cells to the level of the control group at normal temperature (*p* > 0.05).

### 3.7 Intestinal antioxidant capacity

As shown in Table 6, high temperature significantly compromised the duodenal antioxidant status. Relative to the NC group, the HC group showed a 51.2% elevation in MDA content and a 46.9% increase in ROS levels (*p* < 0.05), while duodenal T-AOC activity was reduced by 24.0% (*p* < 0.05). Dietary supplementation with CX effectively ameliorated these oxidative alterations. The 6 mg/kg CX dose was most effective in the duodenum, significantly reducing MDA and ROS levels by 30.2% and 8.5%, respectively, and enhancing T-AOC activity by 29.5% compared to the HC group (*p* < 0.05). Notably, no significant differences were observed in T-AOC and MDA levels between the HCX6 and NC groups (*p* > 0.05).

**Table 6:** The effects of CX-supplemented diets on intestinal antioxidant capacity under heat stress (*n* = 6).

**Item**	**NC**	**HC**	**HCX4**	**HCX6**	**HCX8**	**HCX10**	***p*-Value**
Duodenum MDA (pmol/g)	64.6 ± 1.95^e^	97.7 ± 2.20^a^	79.6 ± 2.90^c^	68.2 ± 2.09^b^	71.01 ± 2.85^d^	84.9 ± 2.36^de^	< 0.001
Duodenum ROS (μg/g)	0.96 ± 0.06^c^	1.41 ± 0.04^a^	1.40 ± 0.04^a^	1.29 ± 0.04^b^	1.29 ± 0.03^b^	1.39 ± 0.04^a^	< 0.001
Duodenum T-AOC (U/ml)	1.25 ± 0.06^a^	0.95 ± 0.03^d^	1.04 ± 0.02^cd^	1.23 ± 0.05^a^	1.19 ± 0.03^ab^	1.13 ± 0.08^bc^	< 0.001
Jejunum MDA (pmol/g)	61.9 ± 2.44^d^	87.0 ± 2.86^a^	84.8 ± 2.28^a^	67.1 ± 1.63^c^	65.8 ± 2.28^cd^	73.9 ± 0.49^b^	< 0.001
Jejunum ROS (μg/g)	0.99 ± 0.08^d^	1.37 ± 0.03^a^	1.25 ± 0.04^b^	1.22 ± 0.03^bc^	1.16 ± 0.01^c^	1.20 ± 0.05^bc^	< 0.001
Jejunum T-AOC (U/ml)	1.44 ± 0.04^a^	0.96 ± 0.05^d^	1.09 ± 0.03^cd^	1.22 ± 0.04^bc^	1.34 ± 0.14^ab^	1.06 ± 0.16^cd^	< 0.001
Ileum MDA (pmol/g)	58.4 ± 2.33^d^	84.7 ± 1.45^a^	83.3 ± 3.40^a^	73.4 ± 3.17^c^	69.8 ± 1.61^c^	78.1 ± 1.76^b^	< 0.001
Ileum ROS (μg/g)	0.97 ± 0.10^c^	1.34 ± 0.02^a^	1.30 ± 0.03^a^	1.11 ± 0.05^b^	1.09 ± 0.03^b^	1.31 ± 0.03^a^	< 0.001
Ileum T-AOC (U/ml)	1.37 ± 0.02^a^	0.96 ± 0.02^c^	1.17 ± 0.05^b^	1.23 ± 0.05^b^	1.36 ± 0.04^a^	1.21 ± 0.04^b^	< 0.001

MDA, malondialdehyde; ROS, reactive oxygen species; T-AOC, total antioxidant capacity. Data are presented as “mean ± SD.” In the same row, values with no letter or the same letter superscripts mean no significant difference (p > 0.05), while values with different letter superscripts mean significant difference (p < 0.05).

As indicated in Table 6, high temperature significantly compromised the jejunal antioxidant status. Relative to the NC group, the HC group showed a 40.5% elevation in MDA content and a 38.4% increase in ROS levels (*p* < 0.05), while jejunal T-AOC activity was reduced by 33.3% (*p* < 0.05). Dietary supplementation with CX effectively ameliorated these oxidative alterations. The 8 mg/kg CX dose demonstrated the best efficacy in the jejunum, significantly reducing MDA and ROS levels by 24.3 and 15.3%, respectively, and enhancing T-AOC activity by 39.6% compared to the HC group (*p* < 0.05). Notably, the MDA and T-AOC levels in the HCX8 group were restored to levels showing no significant difference from the NC group (*p* > 0.05).

As presented in Table 6, high temperature significantly compromised the ileal antioxidant status. Relative to the NC group, the HC group showed a 45.1% elevation in MDA content and a 38.1% increase in ROS levels (*p* < 0.05), while ileal T-AOC activity was reduced by 29.9% (*p* < 0.05). Dietary supplementation with CX effectively ameliorated these oxidative alterations. The 8 mg/kg CX dose demonstrated the best efficacy in the ileum, significantly reducing MDA and ROS levels by 17.6 and 18.7%, respectively, and enhancing T-AOC activity by 41.7% compared to the HC group (*p* < 0.05). Notably, the T-AOC level in the HCX8 group was restored to a level showing no significant difference from the NC group (*p* > 0.05).

### 3.8 Serum antioxidant capacity

As presented in Table 7, high temperature significantly compromised the serum antioxidant status. Relative to the NC group, the HC group showed a 58.1% elevation in MDA content and a 17.5% increase in ROS levels (*p* < 0.05), while serum T-AOC, SOD, and GSH-Px activities were reduced by 13.8, 73.3, and 49.7%, respectively (*p* < 0.05). Dietary supplementation with CX effectively ameliorated these alterations. The 8 mg/kg CX dose demonstrated the best efficacy, significantly reducing MDA and ROS levels by 24.1 and 12.9%, and enhancing T-AOC, SOD, and GSH-Px activities by 12.7, 111.5, and 24.6% compared to the HC group (*p* < 0.05). Notably, the T-AOC and ROS levels in the HCX8 group were restored to levels showing no significant difference from the NC group (*p* > 0.05).

**Table 7:** The effects of CX-supplemented diets on serum antioxidant capacity under heat stress (*n* = 6).

**Item**	**NC**	**HC**	**HCX4**	**HCX6**	**HCX8**	**HCX10**	***p*-Value**
Serum MDA (nmol/ml)	8.09 ± 0.04^c^	12.8 ± 0.45^a^	10.3 ± 0.19^b^	9.75 ± 0.85^b^	9.71 ± 0.54^b^	10.2 ± 0.29^b^	< 0.001
Serum T-AOC (U/ml)	15.0 ± 0.53^a^	13.0 ± 0.34^b^	13.1 ± 0.23^b^	14.6 ± 0.22^a^	14.8 ± 0.26^a^	14.5 ± 0.15^a^	< 0.001
Serum SOD (U/ml)	432 ± 9.64^a^	115 ± 8.49^d^	217 ± 12.5^c^	228 ± 10.8^bc^	244 ± 7.55^b^	221 ± 9.07^c^	< 0.001
Serum GSH-Px (U/mol)	67.9 ± 1.78^a^	34.1 ± 0.79^d^	37.1 ± 0.53^c^	41.5 ± 0.22^b^	42.5 ± 0.40^b^	37.3 ± 0.63^c^	< 0.001
Serum ROS (U/ml)	70.6 ± 1.31^d^	83.0 ± 0.59^a^	78.2 ± 0.90^b^	73.4 ± 0.32^c^	72.3 ± 0.44^d^	78.6 ± 1.11^b^	< 0.001

MDA, malondialdehyde; T-AOC, total antioxidant capacity; SOD, superoxide dismutase; GSH-Px, glutathione peroxidase; ROS, reactive oxygen species. Data are presented as “mean ± SD.” In the same row, values with no letter or the same letter superscripts mean no significant difference (p > 0.05), while values with different letter superscripts mean significant difference (p < 0.05).

### 3.9 Jejunal nutrient transporter expression

**Figure 5:**
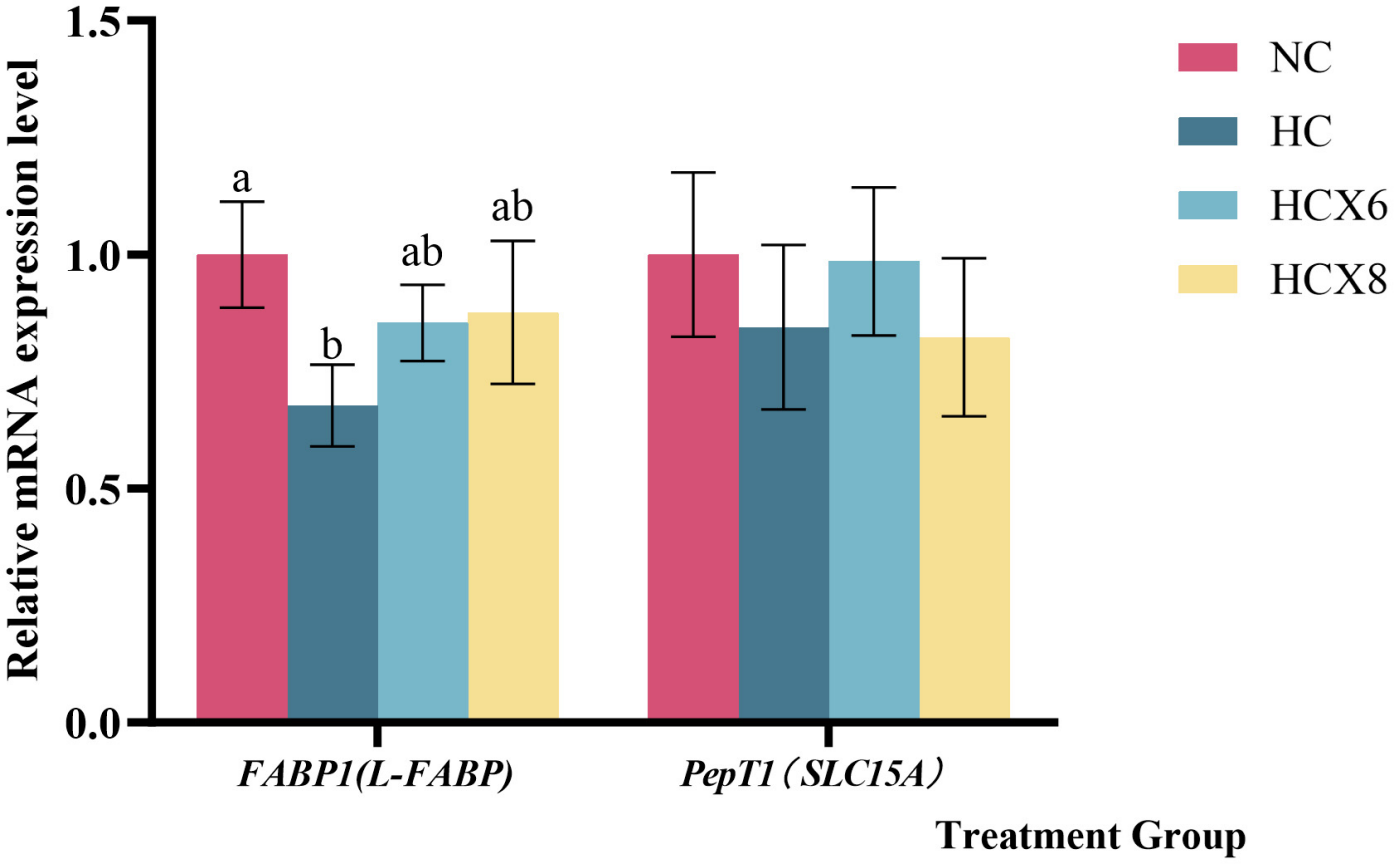
Effects of CX supplementation at varying concentrations on the relative mRNA expression levels of Fatty acid binding protein *FABP1* and small peptide transporter *PepT1* in the Jejunum under heat stress. Values with different letters indicate significant differences (*p* < 0.05), while those with the same letters or no letters indicate no significant differences (*p* > 0.05).

Based on the results of intestinal morphology and apoptosis, this study further detected the mRNA relative expression of *FABP1* and *PepT1* in jejunum epithelial cells of NC, HC, HCX6, and HCX8 groups by qPCR. As illustrated in Figure 5, the relative expression of *FABP1* in the jejunum, was significantly decreased by high temperature (*p* < 0.05). Relative to the NC group, adding 6,8 mg/kg CX at high temperature had no significant effect on *FABP1* expression in the jejunum (*p* > 0.05).

## 4 Discussion

The findings from this research indicated that the high-temperature environment significantly reduced the feed intake and egg production rate of Huaixiang chicken, which was in line with the findings of Kim et al. (22). The mechanism underlying heat stress-induced reduction in egg production primarily involves two pathways: first, inhibition of the hypothalamic feeding center leading to significantly decreased feed intake and limited nutrient supply (23); and second, disruption of protein and lipid metabolic homeostasis (24). Specifically, heat-stress-induced physiological responses, particularly elevated corticosterone levels, enhance systemic protein catabolism (25) while impairing hepatic lipoprotein (including VLDL) synthesis and transport (26). These metabolic alterations directly limit the availability of amino acid substrates for ovalbumin synthesis and reduce delivery of lipid precursors (primarily VLDL-bound) to the ovary, ultimately compromising egg white and yolk formation. As a keto-carotenoid, CX's conjugated double bond structure confers potent free radical scavenging capacity and antioxidant activity (27). Earlier studies have shown that CX as a feed supplement improves the rate of egg production in broilers under thermoneutral conditions (28). Our results revealed that although CX supplementation under elevated temperatures did not affect feed intake, it markedly increased the egg production rate and feed conversion ratio. This intriguing finding indicates that CX's mechanism of action functions independently of both the hypothalamic-pituitary axis and appetite stimulation, but rather by enhancing nutrient utilization and metabolic efficiency. We postulate that the primary mechanism involves the mitigation of heat stress-induced intestinal oxidative damage by CX's potent antioxidant activity. An improved intestinal barrier function and absorptive capacity would allow for more efficient extraction and assimilation of nutrients from the same amount of feed. This hypothesis is robustly supported by our data showing significantly elevated serum levels of total protein, triglycerides, and cholesterol in the CX-supplemented groups. These metabolites serve as essential substrates for egg formation: amino acids are utilized for albumen synthesis in the magnum, while lipid precursors (transported via VLDL) support yolk development in the ovary. The enhanced systemic circulation of these key components thereby directly promotes an elevated rate of egg production. Thus, we propose that CX's antioxidant properties mitigate heat stress-induced intestinal oxidative damage, thereby improving nutrient absorption efficiency. This metabolic optimization increases serum total protein contents and key lipid concentrations (triglycerides and cholesterol), ensuring adequate amino acid supply for ovalbumin production and sufficient lipid precursor delivery for yolk development. Through these mechanisms, CX promotes complete nutrient deposition in egg components, supports normal egg formation, and ultimately enhances egg production rates under thermal challenge.

In this study, high temperature significantly reduced serum levels of TP, TG, TC, HDL-C, and LDL-C, which was consistent with the results of He et al. (29). This metabolic disorder impairs follicle development and yolk deposition efficiency by reducing the synthesis and transport of yolk precursor substances (30). It is crucial to emphasize that, in contrast to its role in human cardiovascular disease, the reduction in LDL-C is particularly detrimental in laying hens due to its unique physiological function. LDL-C serves as the primary circulatory carrier of lipid components (largely derived from hepatic VLDL) for yolk formation in the growing ovarian follicles. The mechanism of heat stress-induced serum nutrient decline mainly involves intestinal morphological damage to reduce nutrient absorption efficiency (31) and down-regulation of nutrient transport-related gene expression to affect nutrient transport (32). Furthermore, previous studies demonstrate that lycopene elevates serum HDL-C (33) and upregulates fatty acid binding protein *FABP2* expression (34), providing important references for exploring the regulation of nutrient metabolism by carotenoids. The study's conclusions demonstrate that dietary supplementation with CX under high-temperature conditions can significantly increase serum TP, TG, TC, HDL-C, and LDL-C. The restoration of LDL-C levels by CX is of paramount importance for reproductive performance, as it ensures adequate delivery of lipid precursors to the ovary for yolk formation, thereby directly supporting yolk development and ultimately contributing to the improved egg production rate observed in our study. Although there has been a lack of specialized research on the effects of CX on serum proteins and lipids at high temperatures, Chen et al. (35) found that CX can considerably raise serum TG, TC, and HDL-C levels in laying hens at room temperature. We speculate that CX may improve intestinal absorption efficiency of important nutrients like TP, TG, TC, HDL-C, and LDL-C by repairing the damage to intestinal villous structure caused by heat stress, increasing the absorption area, and providing the body with a more sufficient nutrient supply.

The morphological integrity of intestinal villi is a fundamental indicator of digestive and absorptive function (36). Optimal structure, characterized by tall villi, shallow crypts, and a high villus-to-crypt (V/C) ratio, is essential for efficient nutrient absorption (37–39), which provides the raw materials necessary for yolk precursor synthesis and egg production. Our results demonstrated that heat stress-induced oxidative stress significantly reduced villus height (VH), V/C ratio, and critically, the VSA across all intestinal segments. This was accompanied by villus atrophy, breakage, and disorganized microvilli in the absorptive cells, aligning with the classic heat stress-induced intestinal injury profile described by Ashraf et al. in broilers (40). The primary driver of this damage is the heat stress-induced persistent accumulation of reactive oxygen species (ROS) in intestinal tissue. Our results confirmed a marked increase in ROS and malondialdehyde (MDA) levels in the serum and intestinal tissues of heat-stressed Huaixiang chickens, indicating a severely compromised antioxidant defense system. This oxidative insult triggers epithelial apoptosis through mitochondrial and lysosomal pathways: ROS-induced oxidative damage to mitochondria initiates a cytochrome c/dATP-dependent caspase-3-mediated apoptotic cascade (41), while concurrent ROS signaling augments lysosomal membrane permeability, facilitating the release of cathepsin B into the cytosol and activating the lysosomal-mitochondrial apoptotic axis (42, 43). Consequently, we observed a significant increase in the apoptosis rate of epithelial cells in the duodenum, jejunum, and ileum, a finding consistent with Liu et al. (44). At the cellular level, absorptive cells exhibited classic apoptotic features, including nuclear membrane shrinkage, blurred cell membranes, and increased apoptotic bodies. The loss of intestinal epithelial cells (IECs), which are central to nutrient absorption, disrupts villus structural integrity (45), which manifests as reduced nutrient absorption area (46), diminished brush border digestive enzyme activity (47), and downregulated expression of nutrient transport carriers (48). The combination of structural damage and cellular loss creates a vicious cycle: the insufficient nutrient supply fails to meet the demands for hepatic synthesis of yolk precursors (35). This nutritional deficiency subsequently hinders follicular maturation and ovulation (49), providing a comprehensive pathological explanation for the observed decline in egg production rate.

Studies have confirmed that carotenoids are bioactive compounds beneficial to intestinal health and contribute to the enhancement of the intestinal structure and morphology in animals (50). Previous research has shown that astaxanthin significantly enhances jejunal VH and V/C ratios in broilers (51), whereas lycopene can improve the intestinal absorption surface area of finishing pigs (52) and rabbits (53). However, it remained unclear whether CX could mitigate heat stress-induced declines in egg production by improving intestinal morphology. Our results address this gap by demonstrating that CX effectively repaired the atrophy and breakage of small intestinal villi, improved villus height (VH) and the villus height-to-crypt depth ratio (V/C), and significantly increased the villus surface area (VSA). Furthermore, CX supplementation helped maintain the structural integrity of microvilli in epithelial absorptive cells. The significant increase in VSA provides quantitative morphological evidence for an enhanced intestinal absorptive capacity, which is pivotal in explaining the subsequent improvement in nutrient absorption efficiency. Importantly, we also observed a significant decrease in the number of apoptotic bodies in the intestinal epithelial absorptive cells of the CX group, with distinct boundaries of the cell membranes. Taken together, these improvements in intestinal morphology and ultrastructure, particularly the reduction in epithelial cell apoptosis, prompted us to investigate the potential underlying mechanisms. Given that the antioxidant properties of compounds like milk thistle powder are known to significantly improve the height of jejunal villi in Leghorn laying hens, thereby enhancing egg production, mainly because of the outstanding antioxidant properties of milk thistle powder (54), and that carotenoids are potent antioxidants (55), we hypothesized that CX exerts its protective effects through enhanced antioxidant capacity. Supporting this hypothesis, our study revealed that dietary supplementation with 6 and 8 mg/kg of CX substantially enhanced the activities of T-AOC, SOD, and GSH-Px in the intestinal tissue and serum of Huaixiang chickens, while reducing the contents of ROS and MDA. While the precise antioxidant mechanism of CX under high temperatures is not fully elucidated, evidence from related carotenoids offers clues. Lycopene (9) and astaxanthin (56) alleviate oxidative stress by activating the nuclear factor E2-related factor 2 (Nrf2) pathway to scavenge reactive oxygen species. We therefore hypothesize that CX may similarly upregulate Nrf2 expression, thereby enhancing the activities of SOD and GSH-Px, efficiently scavenging ROS, and inhibiting the mitochondrial-dependent apoptotic pathway (57). This potential mechanism, however, requires validation in future studies. In conclusion, our findings suggest that CX dietary supplementation mitigates heat stress-induced damage through a multi-faceted approach: it enhances the systemic antioxidant defense system (potentially via the Nrf2 pathway), which in turn reduces oxidative stress and inhibits intestinal epithelial cell apoptosis. This protection allows for the repair of intestinal tissue and the restoration of villus morphology and absorptive function. The consequent improvement in nutrient absorption (evidenced by increased serum total protein and lipid levels) ultimately translates into the recovery of the egg laying rate.

This study further elucidated the mechanism by which heat stress impairs nutrient absorption, specifically through the differential regulation of key intestinal transport genes. We demonstrated that high temperature markedly reduced the expression of *FABP1*—a pivotal regulator of intestinal fatty acid transport (58)—consistent with previous reports (59). This downregulation may be mechanistically linked to heat stress-induced impairment of PPARα signaling, a key transcriptional regulator that directly transactivates *FABP1* expression in the intestine (60), alongside physical damage to the intestinal villi. Notably, dietary supplementation with 6 and 8 mg/kg CX under heat stress conditions significantly upregulated jejunal *FABP1* expression by 25 and 29.4%, respectively, restoring it to levels comparable to the normal temperature group. We propose that carotenoid components in CX may act as ligands or activators of PPARα (34, 50), thereby directly counteracting the transcriptional suppression of *FABP1* induced by heat stress. The restoration of *FABP1* expression, coupled with CX-mediated morphological repair of villi (evidenced by improved VH/CD ratio and VSA), synergistically enhanced intestinal lipid absorption. This increase in intestinal lipid absorption elevates portal circulation fatty acid levels, which are subsequently taken up by the liver and reassembled into VLDL (30). VLDL then serves as the primary vehicle for lipid delivery to developing ovarian follicles (45), where it facilitates yolk deposition and egg formation through receptor-mediated endocytosis. Collectively, these findings provide a coherent mechanistic explanation for the 20.9 and 23.9% increase in egg production rate observed in the 6 and 8 mg/kg CX groups, respectively, compared to the HC group. In contrast, *PepT1* transcription is governed by hypoxia-inducible factor 1α (HIF-1α) (61) and operates independently of the PPARα pathway. Our data align with previous work by Sun et al. (62), confirming that heat stress exerts no significant effect on *PepT1* mRNA levels, which indicates preserved integrity of the HIF-1α-mediated transcriptional machinery under thermal challenge. Since *PepT1* transcription is not impaired, there is no biological need for CX to intervene via transcriptional pathways, which explains why CX does not alter *PepT1* expression. Unlike the expression-dependent activity of *FABP1, PepT1* function is strictly contingent upon the H^+^ electrochemical gradient across the apical membrane of intestinal epithelial cells (63, 64). This gradient is sustained through the coordinated activities of the Na^+^/H^+^ exchanger 3 (NHE3) and Na^+^/K^+^-ATPase (65). Despite unimpaired *PepT1* expression, heat stress disrupts peptide absorption by collapsing this essential H^+^ gradient via two synergistic mechanisms: ROS-mediated oxidative damage that compromises NHE3-dependent H^+^ extrusion, and mitochondrial dysfunction that reduces ATP availability, thereby inhibiting Na^+^/K^+^-ATPase activity (66) and undermining the Na^+^ gradient essential for NHE3 operation. The resultant dissipation of the proton motive force effectively abrogates *PepT1*-mediated peptide uptake. Given that *PepT1* is responsible for the majority of di-/tripeptide uptake, its sustained functional inhibition imposes a ceiling for protein absorption efficiency. Therefore, although CX effectively alleviates intestinal oxidative damage and improves morphology, it cannot overcome the primary biophysical disruption of the proton motive force. This fundamental limitation explains the inability of serum total protein levels to recover fully to those seen under normal temperature conditions.

This study has several limitations. First, mortality was not predetermined as a study endpoint and was therefore not systematically recorded. Although no evident mass mortality occurred during the trial, future studies should include mortality as a key welfare and economic indicator to comprehensively assess the efficacy of canthaxanthin under heat stress. Second, the cyclic heat stress model (8 h/day) employed here, while reflective of diurnal patterns in open-sided housing, may not fully represent the impact of constant heat stress encountered in fully enclosed systems.

## 5 Conclusions

Findings indicate that CX-supplemented diets under high-temperature conditions effectively improved intestinal mucosal structure and nutrient absorption in chickens. Additionally, CX significantly mitigated oxidative damage induced by heat stress and enhanced the laying rate. Based on these findings, the optimal dosage of CX in practical production is 8 mg/kg.

## Data Availability

The original contributions presented in the study are included in the article/supplementary material, further inquiries can be directed to the corresponding authors.
